# DNA-Protective, Antioxidant and Anti-Carcinogenic Potential of Meadowsweet (*Filipendula ulmaria*) Dry Tincture

**DOI:** 10.3390/antiox13101200

**Published:** 2024-10-03

**Authors:** Tsvetelina Andonova, Yordan Muhovski, Elena Apostolova, Samir Naimov, Silviya Mladenova, Iliya Slavov, Ivayla Dincheva, Vasil Georgiev, Atanas Pavlov, Ivanka Dimitrova-Dyulgerova

**Affiliations:** 1 Department of Botany and Biological Education, Faculty of Biology, University of Plovdiv “Paisii Hilendarski”, 4000 Plovdiv, Bulgaria; ts_andonova@uni-plovdiv.bg (T.A.); ivadim@uni-plovdiv.bg (I.D.-D.); 2 Biological Engineering Unit; Life Sciences Department, Walloon Agricultural Research Centre, 5030 Gembloux, Belgium; 3 Department of Molecular Biology, Faculty of Biology, University of Plovdiv “Paisii Hilendarski”, 4000 Plovdiv, Bulgaria; eapostolova@uni-plovdiv.bg (E.A.); naimov0@uni-plovdiv.bg (S.N.); 4 Department of Human Anatomy and Physiology, Faculty of Biology, University of Plovdiv “Paisii Hilendarski”, 4000 Plovdiv, Bulgaria; silviamladenova.sm@uni-plovdiv.bg; 5 Department of Biology, Faculty of Pharmacy, Medical University of Varna, 9000 Varna, Bulgaria; ijelev80@abv.bg; 6 Department of Agrobiotechnologies, AgroBioInstitute, Agricultural Academy, 1164 Sofia, Bulgaria; ivadincheva@abi.bg; 7 Laboratory of Cell Biosystems, Institute of Microbiology, Bulgarian Academy of Sciences, 139 Ruski Blvd., 4000 Plovdiv, Bulgaria; vasgeorgiev@microbio.bas.bg (V.G.); a_pavlov@uft-plovdiv.bg (A.P.); 8 Department of Analytical Chemistry and Physical Chemistry, Technological Faculty, University of Food Technologies, 4002 Plovdiv, Bulgaria

**Keywords:** *Filipendulae ulmariae herba*, dry tincture, phenolic profile (HPLC), in vitro antioxidant, DNA protective, antiproliferative activities

## Abstract

Nowadays, interest in natural antioxidants (especially phenolics) for the prevention of oxidative stress-related diseases is increasing due to their fewer side effects and more potent activity than some of their synthetic analogues. New chemical and pharmacological studies of well-known herbal substances are among the current trends in medicinal plant research. Meadowsweet (*Filipendula ulmaria*) is a popular herb used in traditional medicine to treat various diseases (including rheumatic-, inflammatory- and tumor-related disease, etc.). The dry tincture of *Filipendulae ulmariae herba*, collected from the Bulgarian flora, was analyzed using the HPLC method and bioassayed for antioxidant, antiproliferative and DNA-protective activities against oxidative damage. The HPLC phenolic profile showed 12 phenolics, of which salicylic acid (18.84 mg/g dry extract), rutin (9.97 mg/g de), *p*-coumaric acid (6.80 mg/g de), quercetin (4.47 mg/g de), rosmarinic acid (4.01 mg/g de) and vanillic acid (3.82 mg/g de) were the major components. The high antioxidant potential of the species was confirmed by using four methods, best expressed by the results of the CUPRAC assay (10,605.91 μM TE/g de). The present study reports for the first time the highly protective activities of meadowsweet dry tincture against oxidative DNA damage and its antiproliferative effect against hepatocellular carcinoma (HepG2 cell line). Meadowsweet dry tincture possesses great potential to prevent diseases caused by oxidative stress.

## 1. Introduction

The use of herbal extracts and preparations for medicinal and cosmetic purposes dates back to ancient times [1,2]. Nowadays, interest in natural antioxidants, especially phenolics, for the prevention of oxidative stress-related diseases is increasing due to their fewer side effects and more potent activities compared to some of their synthetic analogues [3,4,5]. New chemical and pharmacological studies of well-known herbal drugs are among the current trends in medicinal plant research. They aim to find new phytochemicals and the different applications of their newly discovered properties.

*Filipendula ulmaria* (L.) Maxim. (syn. *Spiraea ulmaria* L.), commonly known as meadowsweet, is a perennial rhizomatous herb that grows in wet meadows. It belongs to the Rosaceae family, a native species in Western Asia and most of Europe, including Bulgaria [6]. The species is a popular medicinal plant used in traditional medicine for its anti-inflammatory, analgesic, wound-healing, antimicrobial, antitumor, astringent and other biological activities [7,8,9,10,11,12]. Its flowering tops (*Filipendulae ulmariae herba*), included in the European Pharmacopoeia (monograph 04/2013:1868), are used as a herbal substance (whole or cut), due to its contents of essential oils and salicylates [13]. The dried herb is recommended for treating joint diseases, colds, digestive and kidney problems, etc. [14]. A review of scientific information shows that the species has a rich chemical composition, in which various secondary metabolites are present, among which phenolic compounds occupy the largest share [12,15,16,17,18,19]. Flavonoids, phenolic acids and terpenes have been isolated from plant extracts and their fractions, tannins, coumarins, organic acids, etc. [17,18,20,21]. Of the flavonoids in various plant parts (mainly aerial), the following have been found: quercetin, rutin, kaempferol, spiraeoside, isoquercitrin, hyperoside, epicatechin, hyperin, quercitrin, catechin, astragalin, etc. [15,16,18,19,21,22,23,24]. Phenolic acids, such as gallic, salicylic, syringic, ellagic, vanillic, ferulic, chlorogenic, caffeic acid derivatives and others, have been reported to be present, as well as tannins (rugosin B, B1, B2; E, E1, E2), terpenes (*β*-carotene, ursolic acid, pomolic acid), etc. [11,15,21,23,24,25,26,27]. Some of the above-mentioned bioactive metabolites are known for their therapeutic potential and play a key role in biological effects [7,11,17,19,20,26]. Antioxidant activities of different degrees of potency have been found in various extracts, predominantly aqueous, methanolic and acetone, of meadowsweet [15,23,24,27,28]. Some researchers consider the aerial parts of *F. ulmaria* suitable for obtaining ethanol extracts with high antioxidant potential [20,26,29]. Such activity has been reported for both naturally grown and in vitro cultures of the plant species [11]. Studies have reported that an aqueous decoction of the flowers is an effective inhibitor of colorectal carcinogenesis and induce a reduction in the size of tumor growths in experimental animals [30]. *F. ulmaria* flower extract inhibits the proliferation of human lung cancer (NCI-H460), melanoma (A375-C5) and breast adenocarcinoma (MCF-7) cell lines [31]. In complex therapy with cyclophosphamide, the ethanol extract of the aerial parts of the plant enhances its effectivity and reduces the growth of metastases (Lewis lung carcinoma) [32]. In addition to a direct antitumor effect, the herb (methanol extract) has a pronounced ability to reduce side effects in the treatment of cancer with the application of chemotherapeutics, such as cisplatin [16]. *F. ulmaria* extract is reported to be suitable as a functional food with antitumor effects [10]. Another health benefit is the ability of plant fractions to increase the skin’s barrier function under oxidative stress, renewing epidermal cells and enhancing other protection mechanisms [25].

The proven healing potential of *F. ulmaria* is the reason for the enduring interest in this medicinal plant species. Considering the fact that the species in Bulgaria has not been studied phytochemically so far, the aim of the present study was set, namely: to determine the phenolic compounds of the dry tincture of *Filipendulae ulmariae herba* from the Bulgarian flora, as well as its potential for antioxidant and antiproliferative effects and to protect DNA from oxidative stress.

## 2. Materials and Methods

### 2.1. Chemicals, Reagents and Cells Lines

For HPLC and the determination of antioxidant activities and total phenols, reagents of the highest analytical purity were used, supplied by Merck KGaA (Darmstadt, Germany): HPLC-grade solvents (acetonitrile CAS number 75-05-8, methanol CAS number 67-56-1, acetic acid CAS number 64-19-7, and ethanol CAS number 64-17-5); chlorogenic acid (analytical standard) CAS number 327-97-9, vanillic acid (analytical standard) CAS number 121-34-6, gallic acid (analytical standard) CAS number 149-91-7, caffeic acid (analytical standard) CAS number 331-39-5, ferulic acid (analytical standard) CAS number 537-98-4, salicylic acid (analytical standard) CAS number 69-72-7, rosmarinic acid (analytical standard) CAS number 20283-92-5, syringic acid (analytical standard) CAS number 530-57-4, *p*-coumaric acid (analytical standard) CAS number 501-98-4, (+)-catechin (analytical standard) CAS number 154-23-4, (-)-epicatechin (analytical standard) CAS number 490-46-0, rutin (analytical standard) CAS number 153-18-4, kaempferol (analytical standard) CAS number 520-18-3, hesperidin (analytical standard) CAS number 520-26-3; ABTS (2,2′-azino-bis (3-ethylbenzothiazoline-6-sulfonic acid) diammonium salt) CAS number: 30931-67-0, DPPH (2,2-diphenyl-1-picrylhydrazyl) CAS number 1898-66-4, neocuproine CAS number 484-11-7, copper (II) chloride CAS number 7447-39-4, ammonium acetate CAS number 631-61-8, TPTZ (2,4,6-Tris(2-pyridyl)-s-triazine) CAS number 3682-35-7, iron (III) chloride CAS number 7705-08-0, sodium carbonate anhydrous CAS number 497-19-8, and Folin & Chiocalteu’s phenol reagent MDL number MFCD00132625.

The specific reagents for DNA in vitro tests were from: Duchefa Biochemie (Haarlem, The Netherlands)–agarose SPI, CAS number 9012-36-6; TBE buffer, CAS number T1507.0100; and Merck KGaA (Darmstadt, Germany)–Trolox (6-Hydroxy-2,5,7,8-tetramethylchromane-2-carboxylic acid), CAS number 53188-07-1; hydrogen peroxide solution, CAS number 7722-84-1; potassium phosphate dibasic, CAS number 7758-11-4.

In the in vitro experiments to determine the antiproliferative activity, the following cell lines were used: HaCaT (ATCC^®^ № PCS-200-011^™^)–human keratinocytes; SH-4 (ATCC^®^ № CRL-7724^™^)–human melanoma; LnCap clone FGC (ATCC^®^ № CRL-1740™)–prostate cancer; HepG2 (ATCC^®^ № HB-8065™)–hepatocellular carcinoma, obtained from American Type Cultures Collection (ATCC, Manassas, VA, USA). Neutral Red (NR) CAS number 553-24-2; Dulbecco’s modified Eagle’s medium (DMEM) CAS number D5030; antibiotics-streptomycin CAS number 3810-74-0, and penicillin CAS number 87-08-1; Dimethyl sulfoxide (DMSO) CAS number 67-68-5, and fetal bovine serum (FBS) MDL number MFCD 00132239 were from Merck KGaA (Darmstadt, Germany).

### 2.2. Plant Material Collection and Identification

**Figure 1:**
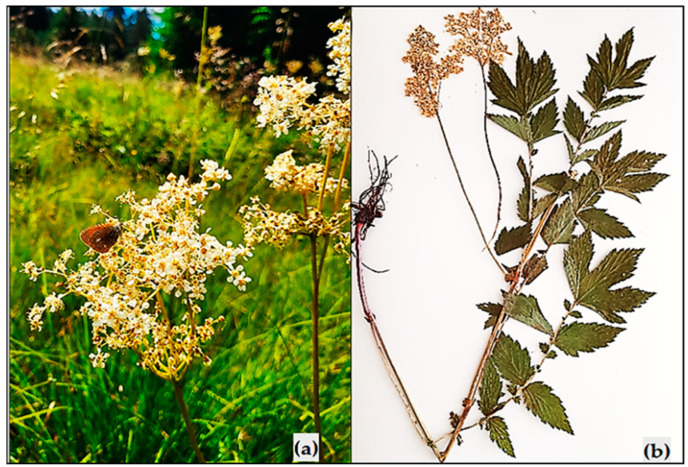
*Filipendula ulmaria*—in the species’ natural habitat (**a**) and herbarium specimen (**b**).

Flowering aerial parts of meadowsweet (*Filipendulae ulmariae herba*) were collected from the Bulgarian flora in the Rhodope Mountains, near the village of Trigrad (N 41°34′15″ E 24°24′23″), from wet meadows, during an active flowering phase in July 2023. An herbarium specimen was deposited under No. 0634003 at the herbarium of the Agricultural University of Plovdiv, Bulgaria (SOA) (Figure 1).

### 2.3. Preparation of the Plant Extract (Dry Tincture, DT)

**Figure 2:**
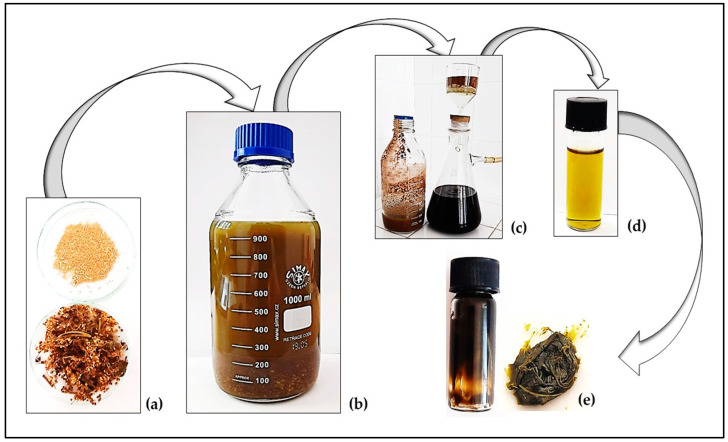
The main steps of the preparation of *Filipendulae ulmaria herba* dry tincture: air-dried herba, crushed and powdered (**a**), maceration with 96% ethanol (**b**); extract filtration (**c**); liquid tincture (**d**); dry tincture obtained (**e**).

The tincture of *Filipendulae ulmariae herba* was obtained through a maceration procedure with 96% ethanol [33]. The air-dried plant material (100 g) was ground with a Retsch knife mill GRINDOMIX GM 200 and placed for ten days in the solvent (1 L) at room temperature, in the dark. Ten parts ethanol to one part of the herbal substance has been described as a suitable procedure for the production of herbal tinctures [33]. The next step was vacuum filtration through an 8–12 µm pore size filter. The resulting extract was vacuum concentrated (BUCHI R-300, Rotavapor, 50 °C and 97 mbar) to obtain the dried tincture and it was stored at −10 °C before chemical and biological analyses. The main steps of the preparation of the *F. ulmaria* herbal extract can be seen in Figure 2, and they correspond to a pharmacopeial procedure for obtaining a dry tincture [34]. Ethanol was selected as the solvent due to it high extraction ability, scanty phytochemical and biological studies of a meadowsweet tincture (96% ethanol), and also it is the most acceptable extractant in the pharmaceutical industry for the preparation of herbal medicines [35]. The DT was dissolved in methanol for HPLS and antioxidant activity assays, in DMSO for determination of antiproliferative activity, and in ethanol for DNA analysis.

### 2.4. Determination of Total Polyphenols and HPLC Phenolic Profile

The total phenolic content was analyzed by the Folin–Ciocalteu method as described elsewhere Krasteva et al. [36]. In brief, 20 µL of extract was used to react with 180 µL ten-time diluted Folin–Ciocalteu reagent in a 96-well plate. After mixing for 2 min, 100 µL of sodium carbonate (7.5%) was added. The reaction was run for 8 min at 37 °C with shaking, and the absorbance at λ = 750 nm against blank (developed in the same way, but without extract) was measured (microplate photometer Multiskan FC, Thermo Fisher Scientific, Waltham, MA, USA). Gallic acid was used to build the standard curve.

The quantification of flavonoids and phenolic acids was performed by using high-performance liquid chromatography (HPLC) as described previously [36]. In brief, 20 µL of filtrated extracts (0.45 µm syringe filters, Thermo Fisher Scientific, Waltham, MA, USA) was injected in Waters 1525 HPLC system (Waters, Milford, MA, USA), at a flow rate of 1.0 mL/min. For compounds separation, a Supelco Discovery HS C18 column (5 µm, 25 cm × 4.6 mm, Merck KGaA, Darmstadt, Germany) was used. A gradient of Solvent A (1% acetic acid in water) and Solvent B (methanol) was applied according to Krasteva et al. [36]. The detection of gallic acid, protocatechuic acid, (+)-catechin, vanillic acid, syringic acid, (-)-epicatechin, p-coumaric acid, salicylic acid, and hesperidin was performed at 280 nm, whereas chlorogenic acid, caffeic acid, ferulic acid, rutin, rosmarinic acid, quercetin, and kaempferol were detected at 360 nm by a Waters 2484 dual λ Absorbance Detector (Waters, Milford, MA, USA). For qualitative analysis, comparison of retention times with those of standards was used. For quantitative analysis, a calibration curve method with external standards was used.

### 2.5. Determination of Antioxidant Activities

Spectrophotometric measurements were performed using a microplate photometer (Multiskan FC, Thermo Fisher Scientific, Waltham, MA, USA). A stock solution of 1.0 mg/mL of extract was prepared in methanol and filtered by a 0.22 µm syringe filter for the antioxidant assays. A 50- and 100-fold dilution of the stock solution was used (20 µg/mL and 10 µg/mL, respectively). Twenty microliters of those extracts (final concentrations of 1.33 µg/mL and 0.66 µg/mL) were used in different antioxidant assays. The results were expressed as micromoles Trolox equivalents per gram dry sample. Standard curves build with Trolox which are linear at concentrations ranging from 50 to 200 µg/mL were used.

#### 2.5.1. DPPH (1,1-diphenyl-2-picrylhydrazyl) Method

DPPH radical scavenging activity was evaluated as described previously [36]. The extract (20 µL) was mixed with 0.1 mM solution of DPPH radical in methanol (280 µL). The reaction was run in a 96-well plate, for 15 min at 37 °C, and the absorbance at λ = 515 nm was measured. A blank sample, without extract was measured as well and used to calculate the percentage of DPPH radical inhibition. A calibration curve (%DPPH inhibition vs. Trolox concentration) was used to calculate the activity of the sample.

#### 2.5.2. ABTS (2,2′-azinobis (3-ethylbenzothiazoline-6-sulphonic acid) Method

The ABTS assay as performed as described previously [36]. Briefly, 20 µL of extract was mixed with 280 µL of freshly generated ABTS radical. The reaction was performed in a 96-well plate, for 15 min at 37 °C, and the absorbance at λ = 734 nm was measured. A blank sample, without extract, was measured as well and used to calculate the percentage of ABTS radical inhibition. A calibration curve (% ABTS inhibition vs. Trolox concentration) was used to calculate the activity of the sample.

#### 2.5.3. FRAP (Ferric Reducing Antioxidant Power) Method

The FRAP assay as performed as described previously [36]. Briefly, 20 µL of extract were allowed to react with 280 µL freshly prepared FRAP reagent in a 96-well plate. The reaction was carried out for 10 min at 37 °C with shaking, followed by measurement of absorbance at λ = 593 nm against a blank (developed in the same way, but without extract). A calibration curve (absorption vs. Trolox concentration) was used to calculate the activity of the sample.

#### 2.5.4. CUPRAC (Cupric Ion Reducing Antioxidant Capacity) Method

The CUPRAC assay was performed as described previously [36]. Briefly, 20 µL of extract was mixed with 70 µL copper dichloride hydrate (10 mM), 70 µL neocuproine (7.5 mM), 70 µL ammonium acetate buffer (1.0 M, pH 7.0), and 70 µL distilled water. The reaction was carried out for 10 min at 37 °C with shaking, followed by measurement of absorbance at λ = 450 nm against a blank (developed in the same way, but without extract). A calibration curve (absorption vs. Trolox concentration) was used to calculate the activity of the sample.

### 2.6. In Vitro Method for DNA Nicking Protective Activity

The DNA protective effect of the plant extract was assessed using supercoiled, covalently closed circular DNA, purified from *E. coli* as described previously [37,38]. One milligram of the preparation was suspended in one mL absolute ethanol, and further diluted (in sterile Milli-Q water) to final concentrations ranging from 100 pg/mL to 10 fg/mL. Ten microliters form each serial diluting was tested in Fenton’s reagent and 400 ng of DNA. All reactions were set to final volume of 20 µL and subsequently incubated at 37 °C for 30 min. Seral dilutions (25, 50, and 100 µg/mL) of Trolox and water were used as positive and negative controls. DNA conformation was then analyzed by 1.0% agarose gel electrophoresis in 0.5× TBE buffer at 120 V for 1 h. The amount of nicked DNA was assessed by densitometry using the inbuild function of a Gel Doc™ EZ Imaging system (Bio-Rad, Hercules, CA, USA). The concentrations of DNA marker bands were used as a reference.

### 2.7. In Vitro Method for Antiproliferative Activity

The NRU assay applied, known as the Neutral Red Uptake in vitro test, is a colorimetric technique used to evaluate cell viability in a laboratory setting [39,40]. This method relies on the ability of living cells to absorb the dye neutral red into their lysosomes. The cells were cultured as a single layer in 25 cm^2^ tissue culture flasks using DMEM high glucose (4.5 g/L) supplemented with 10% FBS and antibiotics. The cultures were kept at 37.5 °C in a humidified environment with 5% CO_2_. For the experiments, cells were plated at a density of 1 × 10^3^ cells in 100 μL of culture medium in each well of 96-well flat-bottomed microplates. They were allowed to adhere for 24 h before being treated with the test substance which was first dissolved in DMSO and then further diluted in the culture medium. Various concentrations of the test substance were applied, after which the cells were incubated for 72 h. A wide range of concentrations was used (from 4 to 1000 μg/mL), after which the cells were incubated for a further 72 h. At the examined concentrations of the extract, DMSO did not exceed 1%.

Following the treatment with Neutral Red medium for 3 h, washing, and application of the ethanol/acetic acid solution, the absorption was measured using a TECAN microplate reader at a wavelength of 540 nm. The results for antiproliferative activity (AA) were presented as percentages.

AA (%) = (1 − (OD_570_ (sample)/OD_570_ (negative control)) × 100 negative control (1% DMSO dissolved in DMEM growth medium)

An indicator of the relationship between the test substance and the specific cell line (normal or tumor) is the selectivity index (SI), calculated as follows:

Selective index (SI): SI = IC50 HaCaT/IC50 Tumor cells

Cisplatin (Sigma-Aldrich, Germany) was used as a positive control.

### 2.8. Statistical Methods

The measurements were recorded in three technical replicates. The obtained data were subjected to statistical analysis and were presented as mean values with the corresponding calculated standard deviations. The statistical significance of the differences between the measured parameters was determined at a 99% confidence level (*p* < 0.01) by one-way analysis of variance (ANOVA) and subsequent post hoc Tukey’s HSD (Honestly Significant Difference) test [41].

## 3. Results

### 3.1. HPLC Phenolic Profile of the Dry Tincture

The obtained dry tincture (DT) was a dense mass with dark green-yellow color (Figure 2e) and its yield was 16.0424 g/100 g dry extract (de). A high content of total polyphenols was measured in the *F. ulmaria* DT—343.34 mg GAE/g, which was a prerequisite to continue with the determination of the individual phenolic composition using an HPLC chromatographic system. Twelve components were identified: 5 flavonoids (4 aglycones and 1 glycoside), and 7 phenolic acids (Table 1, Figure S1).

HPLC analysis of flavonoids showed a predominance of the rutin (glycoside of quercetin) and the flavonol quercetin with amounts of 9.97 mg/g de and 4.47 mg/g de, respectively. The flavanols (+)-catechin, epicatechin, and the flavonol kaempferol had little differences (not significant) in quantities compared to that of the rutin, which were over tenfold lower. Among the phenolic acids identified, salicylic acid stood out with its 18.84 mg/g de. *P*-coumaric acid (6.80 mg/g de), rosmarinic acid (4.01 mg/g de), and vanillic acid (3.82 mg/g de) were also very well represented. Protocatehuic, syringic, and gallic acids were below 1 mg/g de (in range 0.7–0.2 mg/g de).

The performed analysis confirmed the presence of valuable phenolic compounds in *F. ulmaria* DT, which have been proven to be strong natural antioxidants, with beneficial effects for human health, among which six were the best represented, namely rutin, quercetin, salicylic, *p*-coumaric, and rosmarinic acids.

### 3.2. In Vitro Tests for Biological Activities

#### 3.2.1. Antioxidant Activities

The antioxidant capacity of *F. ulmaria* DT was evaluated by four in vitro assays (Table 2). Positive results with high values were reported by all applied methods, as the extract’s potential to reduce cupric ions was the most pronounced (10,605.91 μM TE/g de). The ion reduction ability of the tincture was four times stronger for cupric ions than that for ferric ions. In terms of radical scavenging activity, the difference was in the order of 1.3 times in favor of DPPH•, compared to ABTS•+. The strong antioxidant potential of the studied tincture was confirmed when comparing the data with those obtained for the standards butylhydroxytoluene (BHT) and L-ascorbic acid (vitamin C). The quantitative antioxidant activities (AOA) values of *F. ulmaria* DT by all four methods were higher (more than 2-fold), than those found for butylhydroxytoluene, one of the most widely used synthetic phenolic antioxidants in food and cosmetic products. When comparing the antioxidant power of the tincture with that of the L-ascorbic acid, it can be seen that the plant extract showed very good performance, as even the ability to reduce cupric ions was more pronounced than that exhibited by vitamin C.

The measured high content of total polyphenols, as well as the individual phenolic compounds identified, could explain the demonstrated potent antioxidant effect of the tincture.

#### 3.2.2. In Vitro DNA Protective Capacity

**Figure 3:**
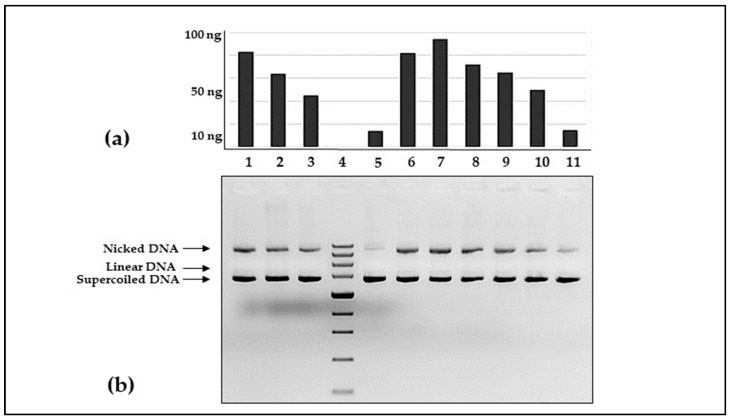
In vitro DNA protective activity of *Filipendula ulmaria* DT: (**a**) relative concentration of nicked plasmid DNA and (**b**) agarose gel electrophoresis. Lines 1–3—different Trolox concentrations (25, 50, and 100 µg/mL); line 4—Zip Ruler 1 Express DNA Ladder (Thermo Scientific, Waltham MA USA, cat. No SM1373); line 5—plasmid DNA input; line 6—negative control; lines 7—11 dilutions of tested extract (10 fg; 100 fg; 1 pg; 10 pg; and 100 pg/mL); The number designations refer to parts (**a**,**b**). The results are from triplicate measurements.

The protective effect of *F. ulmaria* DT on supercoiled DNA was assessed in an in vitro nicking assay experiment. The tested plant extract showed a high level of protection against hydroxyl radicals produced from hydrogen peroxide via Fenton reaction (Figure 3). As expected, a clear correlation between DNA damage and antioxidant concentration was found. The plant’s extract concentration at as low as 100 pg/mL was sufficient to completely prevent DNA damage. The presence of 1 pg/mL tincture provided DNA nicking protection commensurable to the effect of 50 µg/mL Trolox reagent.

The present study proved the high DNA protective activity of the *F. ulmaria* DT, which was due to the presence of compounds with antioxidant properties.

#### 3.2.3. In Vitro Antiproliferative Activity

**Figure 4:**
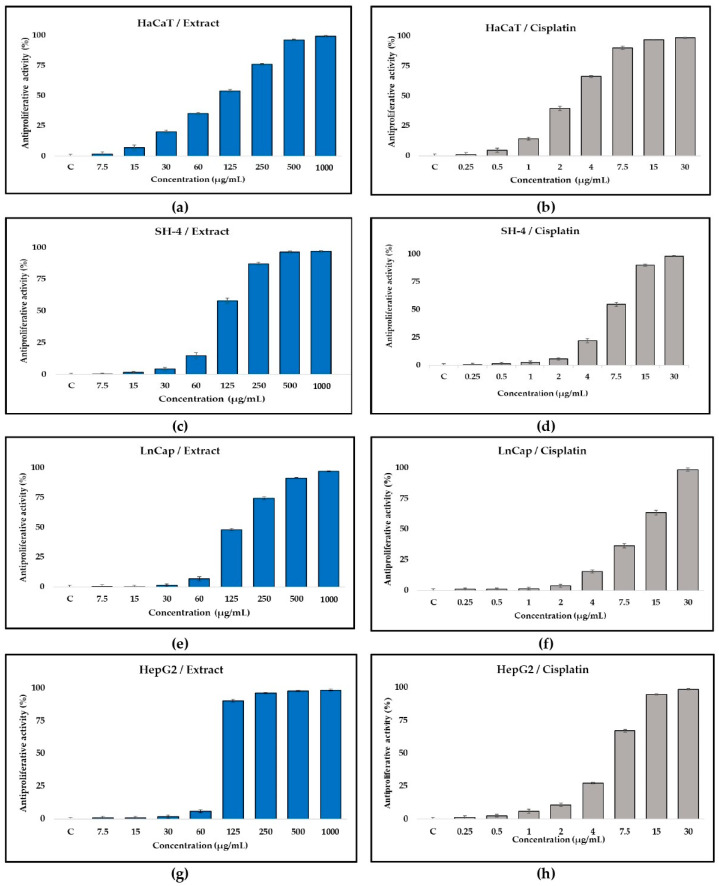
In vitro antiproliferative capacity (%) of *F. ulmaria* DT on (**a**) normal HaCaT (human keratinocytes), and (**c**,**e**,**g**) tumor cell lines LnCap clone FGC (prostate cancer), SH-4 (human melanoma), and HepG2 (hepatocellular carcinoma), respectively. Cisplatin was used as positive control (**b**,**d**,**f**,**h**). Data are presented as means ± standard deviation (SD). The results are from triplicate measurements.

In the in vitro experiments, one normal human and three tumor cell lines were used, respectively: HaCaT (human keratinocytes); SH-4 (human melanoma); LnCap clone FGC (prostate cancer), and HepG2 (hepatocellular carcinoma). The selection of the cell lines was based on their prevalence as tumors and a corresponding comparison between the melanoma tumor line and normal human keratinocytes (Figure 4).

As can be seen from the figure, the antiproliferative activity demonstrates dose dependency only in normal keratinocyte cells, while in tumor cells, the effect of the plant extract (DT) occurs at doses exceeding 125 µg/mL. Measuring the IC_50_ value showed the highest efficacy against hepatocellular carcinoma, with an average of 88.16 ± 1.51 µg/mL, followed by normal keratinocytes and human melanoma (107.54 ± 9.01 µg/mL and 109.65 ± 5.53 µg/mL IC_50_ value, respectively) (Table 3). Prostate cancer tumor cells LnCap clone FGC displayed the lowest sensitivity to the extract (131.81 ± 5.63 µg/mL) among the cell lines tested. There is a direct relationship between the proliferative activity of the cell culture and its sensitivity to drugs with antiproliferative activity. Cytostatics used for antitumor therapy are effective in rapidly developing low-differentiated tumors with a high proliferative index. High proliferative activity of keratinocytes (HaCaT) can be associated with high sensitivity to substances with antiproliferative activity. This is also shown by the obtained mean IC_50_ values for keratinocytes, which are close to the IC_50_ values of tumor cell lines, which usually have a high proliferative potential.

The difference in the observed antiproliferative effect of cisplatin against the cell lines tested confirms the applicability of the method and the reliability of the results obtained for the dry meadowsweet tincture.

An indicator of the relationship between the test substance and the specific cell line (normal or tumor) is the selectivity index (SI). In this scenario, the SI value for both melanoma and prostate tumor lines is low (below 1), suggesting that the extract does not exhibit selectivity towards them (Table 4). Conversely, a well-defined and higher value of 1.22 is observed for hepatocellular carcinoma, indicating the extract’s greater selectivity towards this particular cell line.

The measured SI values of cisplatin, compared to the tincture, did not indicate selectivity against the cell lines tested. 

## 4. Discussion

The data reported in the literature show different amounts of plant extract yield depending on the type of solvent and the plant part used for extraction. According to Sukhikh et al. [28], 70% ethanol extract produced a maximum yield (26.87%) compared to extracts obtained with methanol (23.03%), ethyl acetate (6.23%), or water. They used only plant leaves and applied a methodology different from the current study which explains their higher yield. Apparently, higher temperatures enhance the extraction of the polar components. Similar results were obtained by Pukalskiene et al. [27]. Of the three types of plant extracts they worked with, the one using methanol produced the highest yield (28.61%), higher than the amount found in the current study. For aqueous (8.08%) and acetone extracts (8.53%), they reported twice lower amounts for extract yield.

Content of phenolic compounds (total and individual) in *F. ulmaria* extracts has been reported by various researchers working on the species [15,18,19,21,22,24,28,42]. According to Katanić et al. [15], total polyphenols (methanol extract) expressed as gallic acid equivalents have a value of 249.53 mg GAE/g—a lower measured quantity, compared to the tincture we examined. Other authors have reported amounts of polyphenols for ethanol and aqueous extracts in the scope of 88.0 and 103.0 GAE μg/mL Neagu 2015. Studies focusing on this have found that different drying methods have an impact on the phenolic content (varying in the range of 110–112 mg/g dry biomass) and as the extraction temperature increases from 60 to 100 °C, the concentration of phenols increases [42,43]. For aqueous extracts of the plant species obtained using nanoparticles, a high total phenolic content (about 250 mg GAE/g dry extract) is also observed [44]. According to Yildirim et al. [11], the above-ground portions of *F. ulmaria* (methanol extract) grown in the field have higher total polyphenols (196.80 mg GAE/g dry extract) compared to in vitro grown plants (170.31 mg GAE/g dry extract). Significantly low amounts of total phenols are measured by Pukalskienė et al. [27] in methanol (106.81 mg/GAE g), acetone (40.84 mg/GAE g), and aqueous (22.50 mg GAE/g) meadowsweet extracts. Using the same two solvents (acetone and water) to prepare leaf and flower extracts, other authors report total polyphenols values similar to those in the present study (their higher values are for acetone flower extract—320.5 mg/GAE/g dw) [21]. For methanol extracts of the flowers, Papastavropoulou et al. [45] indicate a significantly lower value of 77.4 mg GAE/g dry sample. Based on the quantitative data for these secondary metabolites in the plant parts (flower, fruit, and leaf) of meadowsweet (59.62–64.65 mg GAE g^−1^), Savina et al. [26] defined them as high, and according to the current study they are even more than five times lower. The difference observed in the amounts of total polyphenols proves that they are influenced by the geographical, climatic, and other specific conditions of the plant species habitat.

The HPLC profile of a flower methanol extract according to Papastavropoulou et al. [45] shows some differences, for instance the predominance of ferulic acid—13.2 mg/100 g ds (dry sample), followed by caffeic acid (6.5 mg/100 g ds), vanillic acid (4.8 mg/100 g ds), and *p*-coumaric acid (4.3 mg/100 g ds), and absence of gallic and syringic acids. They did not find epicatechin and catechin, but indicated the presence of only quercetin (1.5 mg/100 g ds) and rutin (1.3 mg/100 g ds).

A comparative analysis by Sukhikh et al. [28] of the phenolic compounds in leaf extracts (methanol, ethyl acetate and 70% ethanol), proved their greatest diversity in the ethanol extract (9 phenols). In the latter, they also identified rutin, catechin, *p*-coumaric and gallic acids. The same authors reported higher amounts for gallic (0.212 g/kg dw) acid, and lower for coumaric acid (0.038 g/kg dw), compared with the present study. It is interesting that catechin is detected only in the methanol extract and rutin in the ethyl acetate leaf extract, while both flavonoids were identified in the present tincture. Some of the phenolics identified by the current study have also been reported by Bijttebier et al. [19] for *Filipendulae ulmariae herba*. Of the phenolic acids, gallic acid predominated (1340 µg/g), and salicylic acid was measured in amounts almost 30-fold lower than our sample. They also found rutin, quercetin (in 3–4 times lower amounts), and catechin (better represented in 1900 μg/g). Epicatechin (83 μg/g) and kaempferol (64 μg/g) are also part of the phenolic composition of meadowsweet herba. The observed quantitative differences can be attributed to the period of collection of plant material and the different methodology of extract preparation.

In a study on ethanol extracts and fractions from *F. ulmaria* leaves and flowers, Olennikov and Kruglova [18] reported higher amounts of gallic acid compared to our findings (0.48 mg/g and 2.84 mg/g, respectively) Five flavonoids were the same for both studies, but with significant differences in amounts. The data for quercetin (0.67 mg/g) and kaempferol (0.12 mg/g) are several times lower (over 6 times), and for epicatechin, catechin and rutin (3.65–16.21 mg/g) the relationship is reversed. In an ethanol extract (70%) of *F. ulmaria* from Romania, the proportion of quercetin is 132.8 μg/mL [24]. According to the HPLC analysis applied, in addition to the specified metabolite specified, ellagic acid, rutin, quercetin3-*β*-D-glucoside, and chlorogenic acid (106.7–227.7 μg/mL) are significantly present. The authors also identified kaempferol, *p*-coumaric, and rosmarinic acids without specifying exact values to compare with (probably due to low presence, below 2.0 μg/mL). In a hydroalcoholic extract (70%) of crushed aerial parts but obtained with a different methodology and analysis technique (NMR and high-resolution LCMS), other researchers have also identified catechin, kaempferol, quercetin, syringic, gallic and salicylic acids [25].

Gallic acid and salicylic acid were HPLC detected in plant infusion from *F. ulmaria*, in whose chemical composition spiraeoside (56.27 ± 1.03 mg/g) and isoquercitrin (38.44 ± 0.66 mg/g) were the dominant components [23]. High levels of salicylic acid, as well as rutin in the above-ground portions of *F. ulmaria* (naturally- and in vitro-grown) have been recorded by Yildirim et al. [11]. Savina et al. [26] reported higher values for gallic acid in the generative plant parts (5.82% and 4.32% for flowers and fruits, respectively) and lower for leaves and roots. In both studies *p*-coumaric acid is less than 1%. They define salicylic acid as a phenolic acid highly concentrated in flowers (4.51 mg/g^−1^), which, compared to our data, was even four times lower than measured.

With the application of a different methodology (thin-layer chromatography) in the composition of ethanol extracts (70% and 96%), Shilova et al. [20] detected analogous compounds such as quercetin, rutin, salicylic, and gallic acids. Other authors confirm the presence of some of the components identified (rutin, catechin, and gallic acid), but in the composition of a meadowsweet methanol extract [27]. Tannins, flavonoids, and phenolic acids were identified by Sokolova et al. [21] in acetone and aqueous extracts, obtained from leaf and flower parts, but the comparison was not possible due to lack of quantitative values.

Rutin, the flavonoid present in the highest concentration in the studied tincture, is a valuable phytochemical known for a wide range of healing effects. In addition to its strong therapeutic potential (stronger even than synthetically derived dosage forms), an important characteristic of rutin is its harmlessness (in vivo studies prove this for both short-term and long-term intake) [46]. Rutin is indicated as an antitumor agent with different mechanisms of action against various cancers on colon, liver, prostate, and as well leukemia. Quercetin (the second most abundant flavonoid in the dry tincture and a known antioxidant) inhibits the proliferation of colon, ovarian, breast, and other cancers tumor cells. [47,48]. The mentioned above correlates with our discovery of antiproliferative activity against human hepatocellular carcinoma (HepG2 cell line).

Some of the pharmacological effects of the species are caused the salicylic derivatives and precursors of salicylic acid in the phytochemical composition of *F. ulmariae herba* [19]. Usually, a higher content of salicylates is reported for the essential oil composition of the plant species [9,17].

Regarding antioxidant activity, other researchers have also demonstrated the strong ability of *F. ulmaria* to neutralize free radicals. Hydro-alcoholic extracts (60% ethanol) from meadowsweet aerial parts showed higher radical scavenging ability by the ABTS method, compared to DPPH, in contrast to our results for the tincture. Flowers, fruits and upper leaves of meadowsweet exhibit antioxidant power in the range of 130.9–415.7 mg/g ascorbic acid equivalents (AsA), while in the middle/lower leaves and root the effect is weaker [26]. Using the FRAP assay, the quantitative values are the lowest. In Gurita et al. [29], the antioxidant power measured by the DPPH method for ethanol extracts is of significant strength, whose values (72.73–90.42%) are close to those of ascorbic acid used as the standard in the study, as we also found. A comparative analysis by Shilova et al. [20] of the relationship between the concentration of ethanol in hydroalcoholic extracts of the aboveground plant parts and the strength of the AOA shows the highest for 70% and 95% extract, and the lowest for the aqueous, i.e., with an increase in the concentration of ethanol, activity increases. Neagu et al. [24] confirm the above, namely higher activity for ethanol than for aqueous extracts. A better effect (93.49%) was reported for ethanol extracts with a concentration of 3 mg/mL, i.e., at the higher of the tested concentrations, while at a concentration of 1.5 mg/mL, the activity was 80.51%. The AOA for acetone, methanol and aqueous extracts studied by Pukalskienė et al. [27] is lower than in the current study and varies in the range of 0.73–1.99 mg TE/g extract. A seven-fold lower DPPH radical scavenging activity (451.08 μmoL TE/g) was measured by Sukhikh et al. [28] for methanol extract, and they defined it as very strong. Aqueous methanol (62.5%) extracts of the flowers showed an AOA with an IC_50_ of 47.0 mg/L [45].

*F. ulmaria* aerial parts (naturally- and in vitro-grown) analyzed by Yildirim et al. [11] had a high antioxidant effect with an IC_50_ of 205.65 μg/mL and 206.74 μg/mL using the DPPH method, but we could not make a comparison due to different units of measurement. Katanić et al. [15] determined very good IC_50_ values for root extracts (IC_50_ 603.47 μg/mL) and good for aboveground parts. The observed strong ability to scavenge free radicals, according to the latter authors, correlates with the values of total polyphenols and flavonoids, a trend confirmed in the current study.

Extracts of *F. ulmaria* have been tested for various biological activities; however, their effect of protecting DNA molecules from damage by reactive oxygen species have been limited to relaxed DNA [49]. In this article we have demonstrated that quantities of picograms range of *F. ulmaria* ethanol extract are sufficient for complete protection of supercoiled DNA form nicking by hydroxyl radicals. Moreover, studies of Matić et al. [49] showed the absence of genotoxic activity. When compared to the DNA protective effect of plant extracts previously studied by our research team, the protection provided by *F. ulmaria* ethanol extract appeared to be much stronger. The metanalysis of *Ailanthus altissima* [50] and *Koelreuteria paniculata* [37] data shows that the *F. ulmaria* extract overperforms all samples tested before. Its DNA protective potency was estimated to be at list 7 500 times higher than the potency of *A. altissima* and *K. paniculata* extracts regardless of their origin. In the case of *A. altissima,* approximately 750 ng/mL of extract was needed to achieve DNA nicking protection equivalent to the protection of 100 µg/mL of Trolox. The DNA protective activity of herbal extracts rich in antioxidants has been proven for other plant species. Poorna et al. [51] studied the ability of methanol and aqueous extracts of leaves of *Excoecargia agallocha* to reduce DNA damage caused by Fenton’s reagent. According to the authors, the power of the protective effect depends on the concentration, and is significant at 100 µg/µL, comparable to that of catalase and quercetin. The plant species’ aqueous extract also demonstrates protective activity, preserving all three forms of native DNA, indicating its ability to effectively mitigate oxidative stress on biomolecules such as DNA. Using similar DNA nicking tests (hydroxyl radicals generated by Fenton’s reagent) Wang et al. [52] determined the protective activity of DNA for an aqueous leaf extract of *Vernonia amygdalina*. Additionally, the authors note that different extraction methods lead to different effects, with aqueous extracts having a higher antioxidant capacity due to the higher content of polyphenolic compounds.

Antitumor effects of *F. ulmaria* aerial part extracts have been the subject of research in many other articles. In the study conducted by Lima et al. [31], the strong cell growth-inhibiting effect of *F. ulmaria* flower extract on non-small cell lung cancer (NCI-H460) was proven. The extract effectively inhibits the growth of these cells by reducing cell proliferation rather than causing changes in programmed cell death. Katanic et al. [16] present interesting results regarding the use of *F. ulmaria* extracts to protect tissues damaged by cisplatin. After 10 days of administration, the extracts tested were shown to reduce oxidative stress and tissue damage in the liver and kidneys induced by cisplatin, while increasing the antioxidant status of experimental animals undergoing cisplatin treatment. By in vivo experiments with rats, Bespalov et al. [30] demonstrated that meadowsweet extracts have the potential to inhibit methylnitrosourea-induced carcinogenesis. There was a significant decrease in the total number of colorectal tumors in experimental animals. Furthermore, the decoction of meadowsweet flowers exhibited the capability to significantly impede tumor growth in rats following an induced carcinogenesis model involving single *γ*-radiation [53]. The ethanol extract derived from the aerial part of *F. ulmaria* demonstrated a dose-dependent antimetastatic effect on mice with Lewis lung carcinoma in the study conducted by Amosova et al. [54]. Additionally, the combined treatment of animals with cyclophosphamide and meadowsweet extract enhanced the antitumor efficacy of the cytostatic drug. These findings were further supported by the authors’ recent investigation, where even a lower dose (25 mg/kg) of meadowsweet extract increased the antitumor activity in combination therapy with cyclophosphamide [32]. Encapsulated extracts of *F. ulmaria* have been recommended as functional foods with oncoprotective properties [10]. In the present study, for the first time, dry tincture of *F. ulmaria* was tested on HepG2 cell line (human hepatocellular carcinoma) and antiproliferative activity was obtained.

## 5. Conclusions

In conclusion, this is the first report on the chemical composition and biological activities of the medicinal plant *Filipendula ulmaria* from the Bulgarian flora. Dry tincture of the plant substance (*Filipendulae ulmariae herba*) was rich in phenolic compounds. A total of twelve flavonoids and phenolic acids were identified in the tincture, with salicylic acid, rutin, *p*-coumaric acid, quercetin, rosmarinic acid, and vanillic acid being the major ones. The above-mentioned components (proven natural antioxidant and anti-tumor agents) probably play a key role for the high potential of meadowsweet tincture to protect DNA molecules from oxidative damage, as well as the demonstrated antiproliferative effect against HepG2 tumor cell line, reported for the first time by this study. The antioxidant activity of the *F. ulmaria* dry tincture has been proven strong, especially its cupric ion reducing capacity. The findings of this work show that the meadowsweet herbal substance with Bulgarian origin has a very good quantitative and qualitative composition, and great potential for protection from oxidative damage. Herbal tincture could be suitable for incorporation into natural-based cosmetic products with anti-aging effect, dietary supplements, phytopreparations and other medicinal forms to improve human health (for prevention or treatment), warranting further research into its application.

## Figures and Tables

**Table 1:** Total and individual phenolics in *Filipendula ulmaria* DT.

№	Compounds	Content, mg/g de *
**Flavonoids**		
1	Rutin	9.967 ^b^ ± 0.006
2	Quercetin	4.470 ^d^ ± 0.271
3	Kaempferol	0.728 ^e^ ± 0.043
4	(+)-Catechin	0.978 ^e^ ± 0.279
5	(−)-Epicatechin	0.496 ^e^ ± 0.012
6	Hesperidin	nd
**Phenolic acids**		
7	Gallic acid	0.097 ^e^ ± 0.007
8	Protocatehuic acid	0.679 ^e^ ± 0.166
9	Vanillic acid	3.824 ^d^ ± 0.227
10	Syringic acid	0.252 ^e^ ± 0.027
11	*p*-Coumaric acid	6.805 ^c^ ± 0.348
12	Salicylic acid	18.836 ^a^ ± 0.535
13	Rosmarinic acid	4.009 ^d^ ± 0.194
14	Chlorogenic acid	nd
15	Caffeic acid	nd
16	Ferulic acid	nd
**Total phenolic content (Folin–Ciocalteu)**		343.34 ± 13.95 mg GAE/g **

* mg/g de—milligram per gram dry extract; ** mg GAE/g de—milligram gallic acid equivalents per gram dry extract; nd—not detected. Data are presented as means ± standard deviation (SD). The results are from triplicate measurements. Values of the measured features with different small superscript letters are significantly different according to Tukey’s test (*p* < 0.01).

**Table 2:** In vitro antioxidant activity of *Filipendula ulmaria* DT and standard antioxidants (BHT and Vitamin C).

Sample	Antioxidant Activity, μM TE/g *			
	DPPH	ABTS	FRAP	CUPRAC
*F. ulmaria* DT	3249.28 ^b^ ± 49.52	2701.31 ^b^ ± 106.64	3186 ^b^ ± 460.83	10,605.91 ^a^ ± 269.56
BHT	1620.52 ^c^ ± 166.66	1483.81 ^c^ ± 147.03	1634.22 ^c^ ± 183.53	3805.91 ^c^ ± 100.23
L-Ascorbic acid	5844.05 ^a^ ± 247.27	3749.84 ^a^ ± 166.64	5389.89 ^a^ ± 292.52	7826.27 ^b^ ± 275.02

* μM TE/g dw—micromol Trolox equivalent per gram; ABTS radical scavenging assay; CUPRAC—Cupric reducing antioxidant capacity assay; DPPH radical scavenging assay; FRAP—Ferric reducing antioxidant power assay. Data are presented as means ± standard deviation (SD). The results are from triplicate measurements. The means with different small letters a, b, c in superscript in columns are significantly different according to Tukey’s test (*p* < 0.01).

**Table 3:** Antiproliferative activity, values of IC_50_.

	Mean IC_50_ ± SD (µg/mL)	
Cell Lines	*F. ulmaria* DT	Cisplatin *
HaCaT	107.54 ^b^ ± 9.01	2.60 ± 0.14
SH-4	109.65 ^b^ ± 5.53	6.86 ± 0.47
LnCap	131.81 ^a^ ± 5.63	10.67 ± 0.75
HepG2	88.16 ^c^ ± 1.51	5.74 ± 0.13

Data are presented as means ± standard deviation (SD). The results are from triplicate measurements. Values of the measured features with different small superscript letters are significantly different according to Tukey’s test (*p* < 0.01). * Cisplatin was used as positive control.

**Table 4:** Selective index (SI)—IC_50_ determined following 72 h treatment with *F. ulmaria* DT.

	Selective Index	
Cell Lines	*F. ulmaria* DT	Cisplatin *
SH-4	0.98	0.38
LnCap	0.82	0.24
HepG2	1.22	0.45

* Cisplatin was used as positive control.

## Data Availability

The data presented in this study are available on reasonable request from the corresponding author.

## Supplementary Materials

The following supporting information can be downloaded at: https://www.mdpi.com/article/10.3390/antiox13101200/s1, Figure S1: Typical HPLC chromatograms (280 nm and 360 nm) of the phenolic acids and flavonoids in *Filipendulae ulmariae herba* ethanol extract (a) and mixture of standards (b). 1—Gallic acid, 2—Protocatehuic acid, 3—(+)-Catechin, 4—Chlorogenic acid, 5—Vanillic acid, 6—Caffeic acid, 7—Syringic acid, 8—(-)-Epicatechin, 9—*p*-Coumaric acid, 10—Ferulic acid, 11—Salicylic acid, 12—Rutin, 13—Hesperidin, 14—Rosmarinic acid, 15—Quercetin, 16—Kaempherol.
