# Supplementary material for: DNA-Protective, Antioxidant and Anti-Carcinogenic Potential of Meadowsweet (Filipendula ulmaria) Dry Tincture
Source: Antioxidants (Basel). 2024 Oct 3;13(10):1200. doi: 10.3390/antiox13101200 (PMC11504252; doi:10.3390/antiox13101200)
Supplement: Supplementary file 1 [file antioxidants-13-01200-s001.zip › antioxidants-3195393-supplementary.pdf]

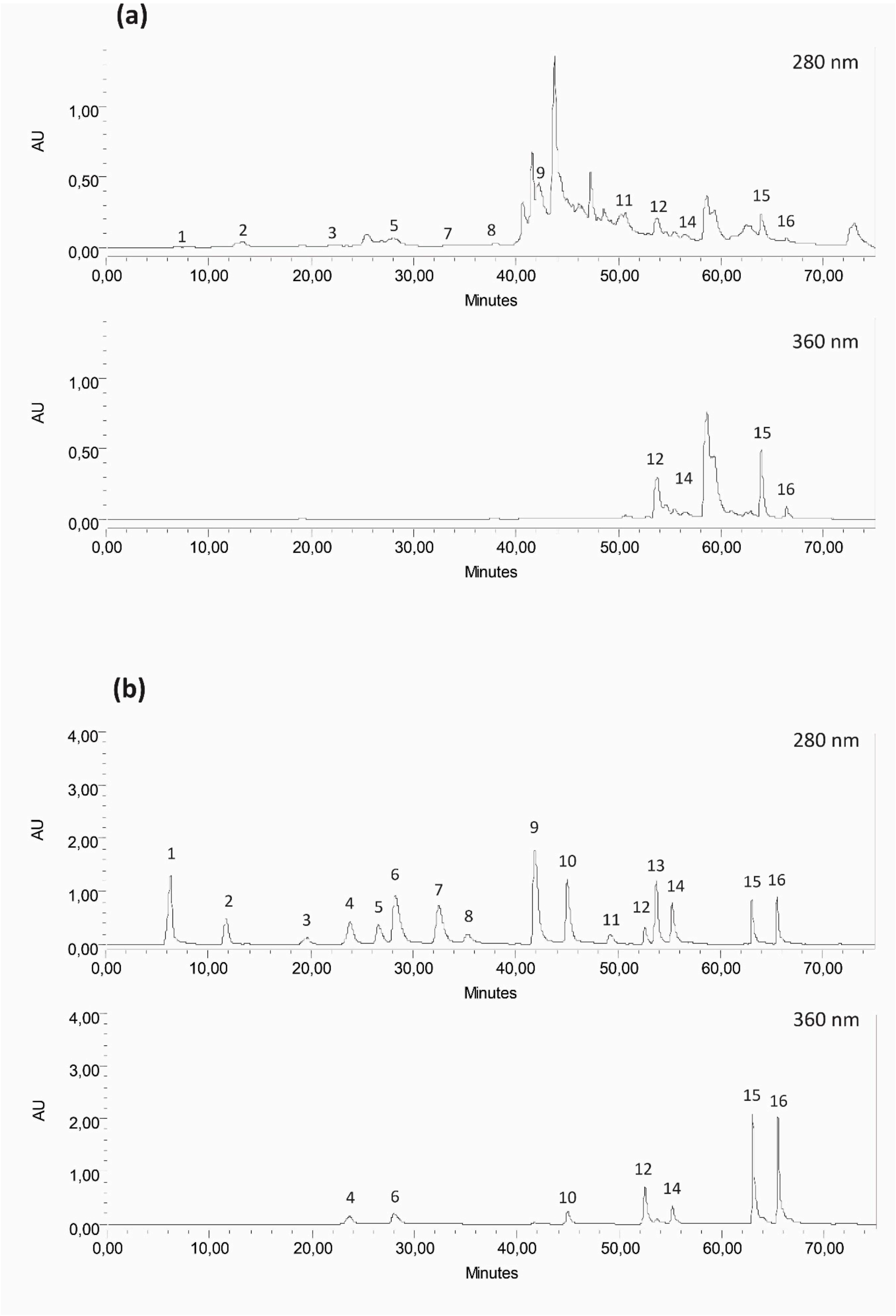
(a)
280 nm
AU
0,00
0,50
1,00
1 2 3 5 7 8 9 11 12 14 15 16
0,00 10,00 20,00 30,00 40,00 50,00 60,00 70,00
Minutes
360 nm
AU
0,00
0,50
1,00
12 14 15 16
0,00 10,00 20,00 30,00 40,00 50,00 60,00 70,00
Minutes
(b)
280 nm
AU
0,00
1,00
2,00
3,00
4,00
1 2 3 4 5 6 7 8 9 10 11 12 13 14 15 16
0,00 10,00 20,00 30,00 40,00 50,00 60,00 70,00
Minutes
360 nm
AU
0,00
1,00
2,00
3,00
4,00
4 6 10 12 14 15 16
0,00 10,00 20,00 30,00 40,00 50,00 60,00 70,00
Minutes

Figure S1. Typical HPLC chromatograms (280 nm and 360 nm) of the phenolic acids and flavonoids in Filipendulae ulmariae herba ethanol extract (a) and mixture of standards (b). 1 – Gallic acid, 2 - Protocatehuic acid, 3 - (+)-Catechin, 4 - Chlorogenic acid, 5 - Vanillic acid, 6 - Caffeic acid, 7 - Syringic acid, 8 - (-)-Epicatechin, 9 - p-Coumaric acid, 10 – Ferulic acid, 11 - Salicylic acid, 12 - Rutin, 13 - Hesperidin, 14 - Rosmarinic acid, 15 - Quercetin, 16 – Kaempherol
